# Understanding Ocular Discomfort and Dryness Using the Pain Sensitivity Questionnaire

**DOI:** 10.1371/journal.pone.0154753

**Published:** 2016-05-03

**Authors:** Wing Li, Andrew D. Graham, Meng C. Lin

**Affiliations:** 1 Vision Science Program, University of California, Berkeley, CA, United States of America; 2 Clinical Research Center, School of Optometry, University of California, Berkeley, CA, United States of America; Sun Yat-sen University, CHINA

## Abstract

**Purpose:**

To utilize the Pain Sensitivity Questionnaire (PSQ) to assess the influence of pain sensitivity on perceptions of ocular discomfort and dryness.

**Methods:**

Subjects completed a battery of questionnaires, including history of ocular and general health, contact lens wear history, the Ocular Surface Disease Index (OSDI) questionnaire, visual analog scale (VAS) 100-point rating scales to assess severity and frequency of average and end of day (EOD) discomfort and dryness, and the PSQ to assess pain sensitivity level. Masked subjects were then instructed to wear one inverted and one normally oriented soft contact lens contralaterally for 30 minutes to induce an inter-eye difference in comfort and dryness sensations. Subjects rated comfort and dryness in each eye on VAS every 5 minutes during contact lens wear. A slit lamp examination was performed to evaluate ocular surface health and to assess contact lens fit.

**Results:**

One hundred and fifty-three subjects (111 females, 42 males) completed the study. In separate models, a higher PSQ score was significantly associated with higher OSDI score (p = 0.002), lower average and EOD comfort (p = 0.005 and 0.001, respectively), and greater EOD dryness (p = 0.04). The minimum (0.14) and maximum (7.14) PSQ scores observed in our subject cohort (i.e., from the subjects who were the least and most sensitive to pain, respectively) corresponded to an estimated difference of 11 points on the OSDI, 20 points on the VAS scale for average comfort, 31 points for EOD comfort and 17 points for EOD dryness. In a mixed effects model, a higher PSQ score was significantly associated with a greater inter-eye difference in comfort (p = 0.013) and dryness (p = 0.010) during CL wear.

**Conclusions:**

Pain sensitivity influences perceptions of ocular discomfort and dryness, and should be taken into account when evaluating subjective assessments of these symptoms.

## Introduction

Due to limitations with diagnostic tests that assess the ocular surface, clinicians often rely on subjective questionnaires to assess and monitor ocular discomfort [1]. Despite such a significant reliance, there has been limited investigation into the factors that influence inter-subject differences in ocular discomfort reported. In response to an identical stimulus to discomfort, individuals can differ greatly in how they perceive it and report it on a questionnaire, [2–6] with people who are more sensitive to pain or discomfort rating the sensation more extremely than would a less sensitive person. Therefore, an instrument that provides some insight into how individuals perceive ocular discomfort could be of benefit in interpreting patient symptomology and influencing treatment decisions.

A validated instrument that measures the level of sensitivity to discomfort could also be useful in examining the often noted discrepancy between clinical signs and patient symptoms of [7–10]. A typical example of this discrepancy can be seen in regards to dry eyes, as it is not uncommon for patients to report dry eye symptoms but lack clinical signs or conversely, present with signs but be asymptomatic, and many studies have found a lack of association between signs and symptoms in dry eye disease [7,8,11–13]. Another example is found in patients with CL discomfort. Although studies have identified a number of factors that are associated with greater discomfort during CL wear (e.g., Asian ethnicity, inferior corneal staining, excessive lens movement, CL surface wettability), there is still significant uncertainty regarding the pathophysiology of CL discomfort [9,10,14–17]. The lack of progress in understanding the relationship between signs and symptoms of ocular discomfort may be due to the failure in recognizing that the level of ocular discomfort experienced is not determined solely by the extent of ocular surface disruption but also by how it is perceived [7,8,18,19].

This is unsurprising, as the perception of ocular discomfort is based on the following neural pathway: (1) the signal (e.g., triggered by an irritant) originates on the ocular surface, (2) is transmitted to the brainstem, (3) then relayed to the limbic system, and (4) finally conveyed to the cerebrum [7,20]. At each step, the signal (and ultimately the perception of ocular discomfort) can either be upregulated or downregulated by nociceptive processing in the brainstem, emotional state in the limbic system, memories of pain in the parietal lobe of the cerebrum and the level of attention given to pain in the frontal lobe of the cerebrum, which are all influenced by a complex interaction of factors[21–25].

This is a similar issue to that which pain researchers have faced in attempting to explain why identical injuries can lead to a diverse range of reported pain or discomfort [2–6]. An insight into this issue was gained with the recognition that the cognitive modulation of pain or discomfort is highly individualized. This biopsychosocial pain model, which as Green explains, states that “pain is ultimately sculpted by complex and dynamic interactions among biological, psychological and sociocultural processes,”[2–4,25–32] suggests that pain sensitivity, defined as how individuals rate painful stimuli, is the most important metric in understanding individual pain perception.[30,33,34] In addition, pain sensitivity has been linked with the level of analgesic use after surgery, the risk of developing chronic pain, and how successful a medical procedure is perceived to be [27,34–37]. In the literature, pain sensitivity has been experimentally measured by determining the level of cold, heat or pressure stimuli a patient could withstand before considering it to be painful [30,33]. In such tests, an individual with higher pain sensitivity would notice pain at a lower stimulus level. The potential of using pain sensitivity to understand ocular discomfort was demonstrated by Vehof et al., but the logistical difficulties of experimentally measuring pain sensitivity have prevented it from being widely studied, as the measurements are time-intensive, expensive, depend on specially trained staff and require inducing pain in healthy subjects [30,33,38].

**Fig 1:**
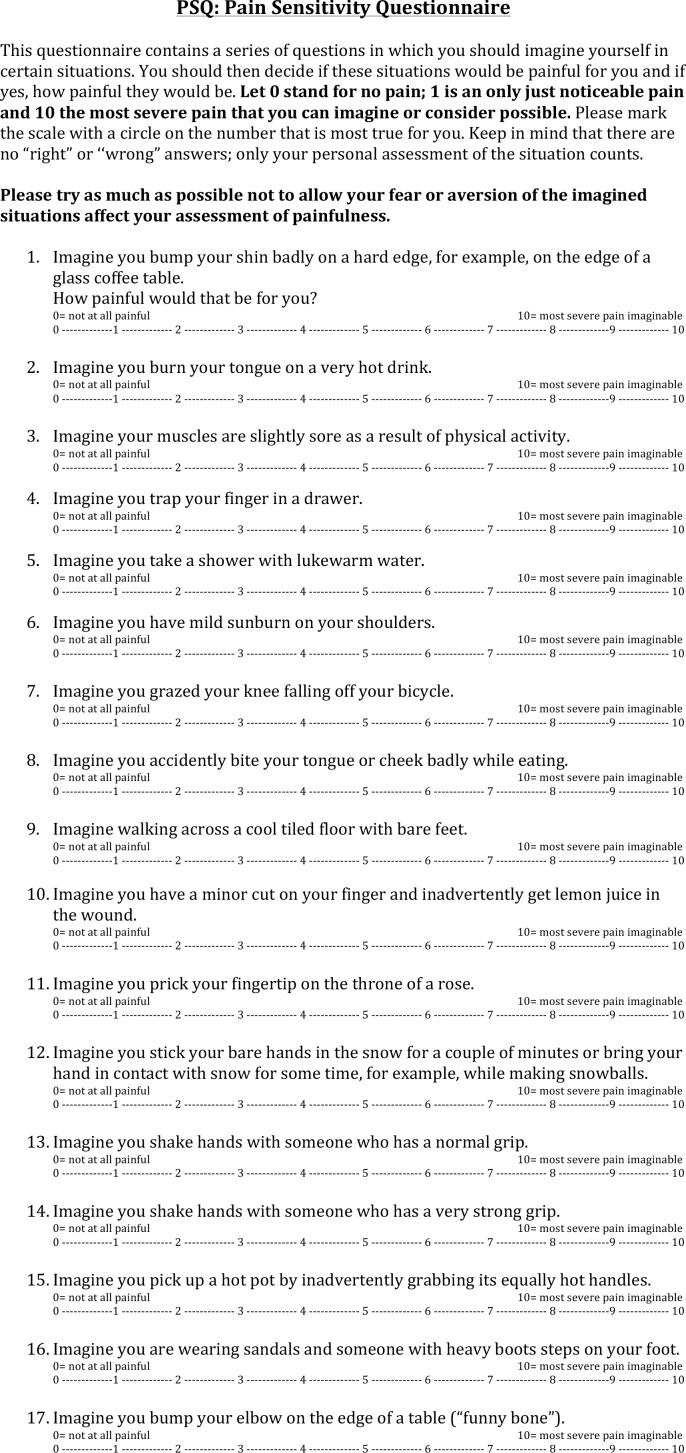
The Pain Sensitivity Questionnaire.

The Pain Sensitivity Questionnaire (PSQ), which was developed by Ruscheweyh et al., may overcome some of these challenges [30]. The PSQ is a self-rating instrument, taking three to five minutes to complete, that asks respondents to imagine themselves in painful situations that are commonly experienced, and to rate the pain they feel they would experience (Fig 1). The questionnaire is simple, requiring no equipment or extensive training, inducing no anxiety in subjects or patients at the prospect of an imminent “pain test”, and being quick to complete even with large numbers of research subjects. The PSQ provides a score that rates pain sensitivity on a 0–10 scale, with a higher score associated with greater pain sensitivity. The PSQ, which has been validated in normal and chronic pain populations, has never been used in ocular surface research [27,33,39–42].

The purpose of this study was to determine if the PSQ score is associated with common subjective instruments for assessing ocular discomfort and dryness symptoms related to dry eye and CL discomfort. We hypothesize that a higher PSQ score (i.e., greater sensitivity to pain) is associated with greater ocular discomfort reported, even after adjustment for any other significant factors. This study could further our understanding of how pain sensitivity may be a factor contributing to the discrepancy between signs and symptoms of ocular discomfort: a patient with greater pain sensitivity may report symptoms in the absence of any clinical signs, while a less sensitive patient may suffer little or no discomfort in spite of visible ocular surface pathology. Furthermore, an awareness of the role of pain sensitivity in patient symptomatology could inform clinician diagnostic and treatment decisions in personalized eye care.

## Methods

### Subjects

Subjects were recruited from the University of California, Berkeley and the surrounding community. Subjects taking systemic or ocular medication, or with a history of systemic or ocular disease or surgery, were excluded from the study. Subjects were also excluded if they were smokers, or currently or previously pregnant. Contact lens wearers (CLW) and non-contact lens wearers (non-CLW) were recruited for the study; non-CLWs were defined as individuals that had never worn CLs before or had discontinued CLs more than one year prior to the study.

The study population consisted of individuals who were of either Asian or Caucasian descent. These two groups of subjects were chosen because previous research has demonstrated inter-ethnic differences in pain sensitivity [29,43], and in both subjective and objective responses to CLs [14,17,44–48]. Individuals were considered to be of Asian ethnicity if they were of Chinese, Korean, Vietnamese or Taiwanese descent, and of Caucasian ethnicity if they were of European descent. Individuals of mixed ethnicity were excluded from the study. Subjects were instructed to refrain from using any eye makeup or eye drops on the day of the visit. Informed consent, with a complete description of the goals, risks, benefits and procedures of the study, was obtained from all participants. This study observed the tenets of the Declaration of Helsinki and was approved by the University of California, Berkeley Committee for Protection of Human Subjects.

### Instrumentation and Procedures

Subjects were administered a baseline questionnaire battery composed of the OSDI, the PSQ, the Dry Eye Flow Chart (DEFC), a set of 100-point visual analog rating scales (VAS) for average and end-of-day (EOD) comfort (0 = poor comfort, intolerable, 100 = excellent comfort, cannot be felt), frequency of discomfort on average and at EOD (0 = Never, 100 = All the time), average and EOD dryness (0 = no sensation of dryness whatsoever, 100 = extremely dry, intolerable), and frequency of dryness on average and at EOD (0 = Never, 100 = All the time). In addition, a demographics and history questionnaire was administered that included items for age, gender, ethnic group (Asian, Caucasian), immigration status (born in the United States or immigrated) and current or past CL wear [17]. The questionnaire battery took approximately 20 minutes to complete and the order of the questionnaires was randomized to minimize any potential bias due to the effects of test fatigue.

In addition to determining whether the PSQ score is significantly related to the aforementioned measures of ocular discomfort, a second goal of the study was to determine whether differences in subjects’ pain sensitivities, as measured by the PSQ, can be shown to partly explain the relatively small differences in comfort and dryness between fellow eyes due to differences in lens fit; therefore, an issue faced during the design of this study was the development of a method to induce such an inter-eye difference. We opted to fit all subjects with a single brand of CL in a single base curve and power (Air Optix Night and Day [B.C. 8.6, Power -1.50 DS]) for both eyes, with one inverted and one normally oriented CL inserted contralaterally based on random assignment. Thus, a relatively small range of differences in discomfort and dryness due solely to differences in lens fit would be induced, eliminating the possibility of more drastic differences we felt may occur in some subjects with different lens designs, surface coatings or soaking solutions. Subjects who indicated a strong baseline preference (on a 5-point Likert scale) for one eye or the other prior to study CL wear were excluded, so that any inter-eye differences in comfort and dryness would be due solely to the different CL fits.

An anterior segment examination under white light was performed prior to CL insertion to ensure there was no evidence of active or pre-existing ocular pathology (e.g., corneal scars, infiltrates, excessive corneal epithelial irritation). Subjects wore the normally oriented and inverted CLs contralaterally for 30 minutes, during which time they completed VAS ratings of comfort and dryness every 5 minutes. Subjects were masked as to which eye received the inverted CL, and were instructed that they could have the CLs removed at any time as they wished. After 30 minutes, a slit lamp examination with fluorescein was performed to assess CL wettability, post-blink movement, tightness and centration. The methods for CL assessment are described further in Tan et al. [14]

### Statistical Methods

The PSQ provides three numerical values: the overall pain sensitivity score (PSQ-Total), and scores for sensitivity to situations with minor (PSQ-min) and moderate (PSQ-mod) pain. The PSQ scores were highly correlated, in agreement with previous studies, and we found through preliminary exploratory analysis (not shown) that the minor pain (PSQ-min) score best reflected the discomfort experienced with dry eye and CL wear [30,33]. Therefore, this analysis will focus on the PSQ-min score; we will refer to the PSQ-min score as the “PSQ score” for the remainder of the manuscript.

After a thorough exploratory and descriptive analysis, baseline questionnaire responses to the OSDI, DEFC, and VAS for average and EOD comfort and dryness (severity and frequency) were modeled as functions of the PSQ score, adjusted for any other significant subject characteristics including age, gender, ethnicity (Asian, Caucasian), immigration status (United States-born, immigrated), history of CL wear, time awake prior to the examination, palpebral aperture size, and presence of grade 2 or greater corneal staining in either eye with white light. Our goal in building such models was to determine whether, after adjusting for any factors that may be related to comfort or dryness outcomes, the PSQ score would remain an additional significant explanatory factor.

After modeling the baseline subjective outcomes, we examined the paired-eye data from 30 minutes of contralateral wear of one normally-oriented and one inverted soft CL, during which time VAS ratings of comfort and dryness for each eye were made by the subject every 5 minutes. We modeled the inter-eye differences (inverted–normally-oriented) in ratings of comfort (IED-C) and dryness (IED-D) as linear mixed effects models, in order to account for the potential within-subject correlations between fellow eyes and over repeated measurements. The candidate explanatory (fixed effects) variables we examined included PSQ score, baseline subject characteristics and baseline symptom ratings, as well as post-wear CL wettability, movement, push-up test tightness, and decentration.

For both the baseline and post-CL wear analyses, the best models were selected based on consideration of F-test p-values, examination of residual and other diagnostic plots, and comparison of the log-Likelihood for nested models or Akaike’s Information Criterion for non-nested models. A subset of subjects felt that the inverted CL was at times more comfortable than the normally oriented CL; because we were not testing hypotheses about inverted-vs.-normally-oriented CLs, but rather simply inverting one lens to create some difference in subjective sensation, we elected to model the absolute values of the IED-C and IED-D. In addition, many subjects found it difficult to provide ratings of comfort or dryness during the initial period of CL settling, which was reflected in excessively high within- and between-subject variability in the first 10 minutes; we therefore elected to analyze subject ratings made between 10 and 30 minutes post-insertion, after the lens had settled. Finally, in order to better approximate normality, we modeled both IED-C and IED-D on the natural log scale.

## Results

### Subject Characteristics

A sample of 168 subjects was initially recruited for the study. Fourteen subjects did not complete the study due to pre-existing corneal scar, mixed ethnicity, or a strong baseline comfort or dryness preference for one eye over the other. One subject was unable to complete all study measurements due to inability to tolerate 30 minutes wear of the study CLs. Further details on the reasons for disqualification and dropout are provided in Table 1 [49]. A total of 153 subjects (40 male, 113 female) with a mean (SD) age of 22.6 (3.4) years (range: 18–34 years) successfully completed the study. The study cohort was composed of 91 Asians and 62 Caucasians; 114 subjects were born in the United States and 39 subjects immigrated to the United States (85% of subjects who immigrated were Asian). Ninety subjects were experienced CLW and 63 were non-CLW.

**Table 1:** Disposition of all recruited subjects at end-of-study.

Subjects Recruited	168
**Failed to Meet Eligibility Criteria**	**8**
Mixed Ethnicity	4
Pre-existing Corneal Scar	2
History of Iritis	1
Unable to Document Recent Eye Exam	1
**Disqualified**	**6**
Unable to Insert CLs	3
Strong Baseline Comfort Preference	3
**Dropouts**	**1**
Unable to Complete 30min CL Wear	1
**Total Failing to Enter and Complete Study**	**15**
**Total Successfully Completing Study**	**153**

### PSQ Score

**Fig 2:**
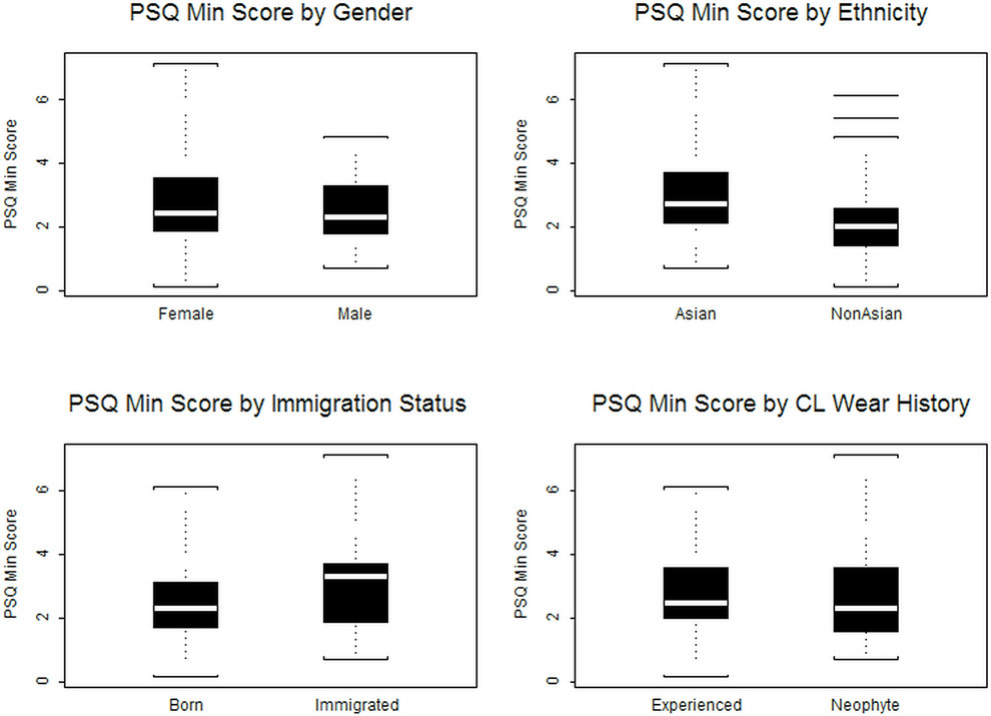
PSQ score stratified on gender, ethnicity, immigration status, and CL wearing history.

The mean (SD) PSQ score was 2.7 (1.3) with a range of 0.1 to 7.1. Fig 2 depicts the PSQ scores stratified on gender, ethnicity, immigration status and CL history. There was no significant difference (p = 0.229) in mean PSQ score between men (2.5) and women (2.8), nor was there a significant difference (p = 0.331) between CLW (2.8) and non-CLW (2.6). Asians had a higher mean PSQ score (3.0) than did Caucasians (2.3), indicating significantly greater pain sensitivity on average among Asians (p<0.001). Subjects who immigrated to America also had a higher mean PSQ score (3.1) than those born in America (2.6; p = 0.021).

### Baseline Questionnaire Response

Descriptive statistics for the baseline questionnaire responses are shown in Table 2. In multivariable linear mixed effects models (Table 3), a higher OSDI score was significantly associated with higher PSQ score (p = 0.005), as well as with female gender (p = 0.016) and Caucasian ethnicity (p = 0.004). There was an estimated 11 point greater OSDI score for the highest (7.1) vs. the lowest (0.1) PSQ scores observed. Lower average comfort was significantly associated with higher PSQ score (p = 0.005), as well as with CLW (p = 0.009). There was an estimated 20 unit lower average comfort for the highest vs. the lowest PSQ scores observed. A greater frequency of discomfort on average was significantly associated with CLW (p = 0.015) and a higher PSQ score (p = 0.009), with an estimated 17 unit higher rating for the highest vs. the lowest PSQ scores observed. Lower EOD comfort was also significantly associated with CLW (p<0.001) and a higher PSQ score (p = 0.001), with an estimated 31 unit lower EOD comfort rating for the highest vs. the lowest PSQ scores observed. A greater frequency of EOD discomfort was significantly associated with being a female (p = 0.009) and CLW (p<0.001), but not significantly associated with PSQ score (p = 0.379).

**Table 2:** Descriptive statistics for baseline questionnaire responses.

	Min	Max	Median	Mean	SD
**PSQ Score**	0.14	7.14	2.43	2.69	1.25
**OSDI**	0.00	45.83	6.25	8.63	8.60
**DEFC**	1	5	2	2.4	1.4
**Avg Comfort**	27	99	87.0	81.3	16.9
**Avg Discomfort Freq**	0	75	9.0	13.7	15.8
**EOD Comfort**	6	99	75.0	69.8	25.0
**EOD Discomfort Freq**	0	99	15.0	25.7	27.3
**Avg Dryness**	0	72	12.0	18.7	19.7
**Avg Dryness Freq**	0	75	9.0	16.3	18.3
**EOD Dryness**	0	87	18.0	27.2	26.6
**EOD Dryness Freq**	0	90	15.0	25.8	27.5

**Table 3:** Separate multivariate models showing the associations between subject characteristics and subjective responses to the baseline questionnaires. The arbitrary reference groups for Gender, Ethnicity and CLWHx (CL Wear History) were Female, Asian and Experienced, respectively. A higher value in average or EOD comfort is associated with greater comfort. A higher value in average or EOD dryness is associated with greater dryness.

Outcome	Intercept	PSQ	Gender: Male	Ethnicity: Caucasian	CLWHx: neophytes
**OSDI**	3.18	1.59 (p = 0.002)	-3.25 (p = 0.016)	3.34 (p = 0.004)	
**Avg Comfort**	85.93	-2.81 (p = 0.005)			7.02 (p = 0.009)
**Avg Discomfort Frequency**	9.78	2.41 (p = 0.009)			-6.15 (p = 0.015)
**EOD Comfort**	74.10	-4.33 (p = 0.001)			17.94 (p = <0.001)
**EOD Discomfort Frequency**	32.87	0.65 (p = 0.379)	-11.08 (p = 0.009)		-14.54 (p = <0.001)
**Avg Dryness**	25.27	0.41 (p = 0.181)		-7.91 (p = 0.020)	-10.83 (p<0.001)
**Avg Dryness Frequency**	23.34	0.07 (p = 0.251)		-8.75 (p = 0.006)	-8.97 (p = 0.002)
**EOD Dryness**	31.38	2.32 (p = 0.040)	-8.01 (p = 0.020)		-20.33 (p = <0.001)
**EOD Dryness Frequency**	-10.43	1.89 (p = 0.242)	-10.13 (p = 0.006)		-21.11 (p = <0.001)
**DEFC Score**	3.19	0.03 (p = 0.069)		-0.45 (p = 0.047)	1.62 (p<0.001)

Higher average dryness severity was significantly associated with being Asian (p = 0.020) and CLW (p<0.001), but not with PSQ score (p = 0.181). A greater frequency of dryness on average was also significantly associated with being Asian (p = 0.006) and CLW (p = 0.002), but not with PSQ score (p = 0.251). Higher EOD dryness severity was significantly associated with higher PSQ score (p = 0.041), as well as with female gender (p = 0.020) and CLW (p<0.001). There was an estimated 16 unit higher EOD dryness severity rating for the highest vs. the lowest PSQ scores observed. A greater frequency of EOD dryness was significantly associated with greater age (p = 0.001), being female (p = 0.006) and CLW (p<0.001), but not with PSQ score (p = 0.242). A higher DEFC score was significantly associated with being Asian (p = 0.047) and CLW (p<0.001), and although the PSQ score approached significance at the α = 0.05 level (p = 0.069) the effect size was clinically insignificant, with an estimated difference of 0.2 units on the 5-unit DEFC scale between the highest and lowest PSQ scores observed.

### Subjective Response During 30 min Contact Lens Wear

Descriptive statistics for the CL fitting characteristics are shown in Table 4. There was no significant difference in wettability between the inverted and normally oriented CLs (p = 0.893). The inverted CL demonstrated more movement after a blink than the normally oriented CL (p<0.001). The inverted CL, on average, showed less lens tightness than the normally oriented CL (p<0.001).

**Table 4:** Mean (SD) and paired t-test p-values for fitting characteristics of the normally-oriented and inverted CLs.

	Inverted CL	Normally-Oriented CL	p-value
**Wettability**	3.59 (0.43)	3.59 (0.44)	0.893
**Movement (in mm)**	0.62 (0.47)	0.31 (0.26)	<0.001
**Tightness**	41.1 (8.5)	52.2 (9.4)	<0.001

There was a decrease in IED-C when comparing the values at 10 and 30 minutes post-insertion (14.0 vs. 11.6, respectively; p = 0.02) but with an estimated difference in VAS rating of less than 3 units on the 100-point scale, it was not clinically significant. There was no significant difference in IED-D when comparing the values at 10 and 30 minutes post-insertion (6.5 vs. 6.3, respectively; p = 0.73). Therefore, in comparing VAS ratings to PSQ scores, the means of the IED-C and IED-D over the twenty-minute measurement period were used for the reminder of the analysis.

Age, gender, ethnicity, immigration status, CLW history, time awake, CL wettability and movement were all found not to be significantly related to the IED-C. The linear mixed effects model showed that a greater comfort difference between fellow eyes was significantly associated with a higher PSQ score (p = 0.013). There was an estimated 7 unit increase in the IED-C rating for the highest vs. the lowest PSQ scores observed.

Gender, ethnicity, CLW history, time awake, CL wettability, movement, and tightness were all found not to be significantly related to the IED-D. The linear mixed effects model showed that a greater dryness difference between fellow eyes was significantly associated with a higher PSQ score (p = 0.010). There was an estimated 7 unit increase in the IED-D rating for the highest vs. the lowest PSQ scores observed.

## Discussion

In this study we found that the PSQ provides a clinically relevant insight into the perception of symptoms of ocular dryness and discomfort. Examining the statistical models, the PSQ score appears to have a significant independent effect on subjective ratings of ocular comfort and dryness, even after adjusting for significant subject demographic and ocular characteristics. As pain sensitivity is based on how painful stimuli are rated, it is not surprising that the PSQ was primarily associated with the severity and not the frequency of ocular discomfort and dryness. We believe that with further work, the PSQ could be employed to provide a deeper insight into ocular discomfort and dryness. As a key example, one of the most confounding aspects of dry eye is the sporadic and unreliable correlation between signs and symptoms of dry eye [7,8]. It is not uncommon for individuals to have the same clinical presentation of dry eye but have vastly different OSDI scores; conversely, very similar OSDI scores can be observed with vastly different clinical signs. Such discrepancies may be explained in part by pain sensitivity either amplifying (in an individual with high sensitivity to pain) or weakening (in an individual with low sensitivity to pain) the perception of dry eye symptoms. A patient with greater pain sensitivity may suffer symptoms of discomfort when ocular surface pathology that could lead to such symptoms is sub-clinical. A less sensitive patient may report no symptoms at all, even when the clinician can clearly identify signs of desiccation and damage to the ocular surface.

At the current time these results are suggestive only, as the purpose of this study was not to directly examine the relationship between DE signs and symptoms, but to determine whether the PSQ could be used to quantify the effect of individual pain sensitivity on ratings of subjective symptoms. Further suggestion of the potential for utilizing the PSQ in future studies can be seen with CL discomfort in this study, for which the PSQ score was the only significant explanatory variable (no CL fitting characteristics were found to be significant). With the PSQ score now established as an independent, significant explanatory factor in our models of several different subjective assessments of ocular discomfort and dryness, future work will include examining the relationship between signs and symptoms in subjects with a wide range of pain sensitivities.

Although Vehof et al. reached a similar conclusion to this study, the logistical challenges of experimentally measuring pain sensitivity as was done in that study limit its clinical and research utility [38]. Experimentally measuring pain poses many issues and it requires the development of a complex plan to measure pain sensitivity, accounting for the type of pain modality used (heat, cold or pressure) and specifications for testing (i.e., strength, placement and timing of stimuli delivery) [30,33]. In addition, inducing pain or the fear of pain-inducement can have a significant cognitive effect on subjects, potentially confounding how they perceive an irritant or answer subjective questionnaires [50–52]. The PSQ overcomes many of these challenges and the noted advantages of the PSQ over experimental pain sensitivity assessments could make it a useful tool in our efforts to better understand ocular symptomatology.

An acknowledgment of the role that pain sensitivity has in influencing ocular discomfort is important because of the limited development in treatment options for dry eye (Restasis® is the only FDA-approved medication for dry eye), which is partially due to the lack of association between signs and symptoms of dry eye [53]. Fifteen companies have sought and failed to get FDA approval for their dry eye drugs; several drugs, most recently Eleven Biotherapeutics’ EBI-005, were unable to pass Phase 3 clinical trials due to inability to show an improvement in signs *and* symptoms. Meeting both prerequisites was not required when Restasis® was FDA approved, but now fulfilling these two criteria is hampered by low repeatability and poor correlation with symptoms seen in current diagnostic tests [53–56]. Even if test repeatability was improved, there may still be a discrepancy between signs and symptoms due to the impact that pain sensitivity has on their relationship, as suggested by this study. Further, as the minimal clinically important difference for the OSDI ranges from 7.0 to 9.9, which is within the effect size seen in this study (11 points, when comparing the subjects with the least and most sensitivity to pain), this suggests that pain sensitivity should be used as a corrective factor when assessing improvements of signs and symptoms in future clinical trials [57].

This study found that the PSQ was associated with factors such as ethnicity (Asians having greater pain sensitivity) and immigration status (immigrants having greater pain sensitivity), which is in agreement with previous studies [29,58,59]. Gender was not found to be associated with PSQ score, which is consistent with other studies [30,33]. In this study, subjects of European-Caucasian descent were associated with a greater OSDI score, which is surprising as subjects of Asian descent were associated with greater EOD dryness and because Asians have been reported to have a greater prevalence of dry eye [60,61]. The discrepancy may be due to sampling variation but it may also be important to consider the inherent difference between the two questionnaires. The EOD dryness VAS consists of one question, “How would you rate the dryness of each eye at the end of the day?” This is in contrast to the OSDI, which has twelve questions that show significantly greater linguistic complexity compared to the VAS. It is possible that Asians, with a third being immigrants, may respond differently (i.e., report less dryness) in a complicated questionnaire compared to a simpler one. This finding highlights the need for further improvements in our understanding of inter-ethnic differences in dry eye.

Comparing subjects with the least and greatest sensitivity to pain (as measured by the PSQ), the inter-eye differences in comfort and dryness were estimated to be approximately 7 points on the 100-point VAS, which is a relatively small but clinically significant difference. Nevertheless, it is possible that this study may offer clues as to why some patients, after years of being asymptomatic CL wearers, suddenly become symptomatic, even without evident clinical signs. The risk of developing dry eyes and CL intolerance increase with age, likely due to alterations to the tear film and ocular surface that occur over time [62,63]. It is possible that minor alterations to the tear film/ocular surface, which may not be considered clinically significant, that occur with age cause symptoms to be magnified in individuals with greater pain sensitivity, leading to CL dropout. This is supported by studies that have found no difference in tear film properties between symptomatic and asymptomatic CLW; the exception being conflicting reports on patients with lid wiper epitheliopathy and patients with conjunctival folds [15,64,65]. The results from this study suggest that a cross-sectional study—and eventually a longitudinal study—is warranted to determine if increased pain sensitivity is a risk factor for the discontinuation of CL wear.

## Conclusions

Using the PSQ, we were able to show that pain sensitivity was related to perception of ocular comfort and dryness. Additionally, pain sensitivity was found to be associated with the subjective assessment of inter-eye differences in comfort and dryness during CL wear. The results suggest that pain sensitivity must be considered when interpreting subjective responses to symptom-related questionnaires. Pain sensitivity differences may also offer a partial explanation for the discrepancy seen between the signs and symptoms of ocular discomfort, including dry eye and CL intolerance or dissatisfaction.

## Supporting Information

S1 Dataset Entire dataset from the study. (XLS)

## References

[1] Baudouin C, Aragona P, Van Setten G, Rolando M, Irkeç M, Benítez del Castillo J, et al Diagnosing the severity of dry eye: a clear and practical algorithm. Br J Ophthalmol. 2014;98(9):1168–76. 10.1136/bjophthalmol-2013-304619 24627252 PMC4145432

[2] Nielsen CS, Staud R, Price DD. Individual differences in pain sensitivity: measurement, causation, and consequences. J Pain. Elsevier Ltd; 2009 3;10(3):231–7. 10.1016/j.jpain.2008.09.010 19185545

[3] Fillingim RB. Individual differences in pain responses. Curr Rheumatol Rep. 2005 9;7(5):342–7. 16174481 10.1007/s11926-005-0018-7

[4] Gatchel RJ, Peng YB, Peters ML, Fuchs PN, Turk DC. The biopsychosocial approach to chronic pain: scientific advances and future directions. Psychol Bull. 2007 7;133(4):581–624. 17592957 10.1037/0033-2909.133.4.581

[5] Greenspan JD, Craft RM, LeResche L, Arendt-Nielsen L, Berkley KJ, Fillingim RB, et al Studying sex and gender differences in pain and analgesia: a consensus report. Pain. 2007 11;132 Suppl: S26–45. 17964077 10.1016/j.pain.2007.10.014 PMC2823483

[6] Kim H, Neubert JK, San Miguel A, Xu K, Krishnaraju RK, Iadarola MJ, et al Genetic influence on variability in human acute experimental pain sensitivity associated with gender, ethnicity and psychological temperament. Pain. 2004 6;109(3):488–96. 15157710 10.1016/j.pain.2004.02.027

[7] Rosenthal P, Borsook D. The corneal pain system. Part I: the missing piece of the dry eye puzzle. Ocul Surf. 2012 1;10(1):2–14. 10.1016/j.jtos.2012.01.002 22330055

[8] Rosenthal P, Baran I, Jacobs DS. Corneal pain without stain: is it real? Ocul Surf. 2009 1;7(1):28–40. 19214350 10.1016/s1542-0124(12)70290-2

[9] Nichols JJ, Willcox MDP, Bron AJ, Belmonte C, Ciolino JB, Craig JP, et al The TFOS International Workshop on Contact Lens Discomfort: executive summary. Invest Ophthalmol Vis Sci. 2013 10;54(11):TFOS7–13. 10.1167/iovs.13-13212 24058135 PMC4686219

[10] Young G, Chalmers R, Napier L, Kern J, Hunt C, Dumbleton K. Soft contact lens-related dryness with and without clinical signs. Optom Vis Sci. 2012 8;89(8):1125–32. 10.1097/OPX.0b013e3182640af8 22820475

[11] Labbé A, Wang YX, Jie Y, Baudouin C, Jonas JB, Xu L. Dry eye disease, dry eye symptoms and depression: the Beijing Eye Study. Br J Ophthalmol. 2013 11;97(11):1399–403. 10.1136/bjophthalmol-2013-303838 24013959

[12] Nichols KK, Nichols JJ, Mitchell GL. The lack of association between signs and symptoms in patients with dry eye disease. Cornea. 2004 11;23(8):762–70. 15502475 10.1097/01.ico.0000133997.07144.9e

[13] Mizuno Y, Yamada M, Miyake Y. Association between clinical diagnostic tests and health-related quality of life surveys in patients with dry eye syndrome. Jpn J Ophthalmol. 2010 7;54(4):259–65. 10.1007/s10384-010-0812-2 20700790

[14] Truong TN, Graham AD, Lin MC. Factors in Contact Lens Symptoms: Evidence from a Multistudy Database. 2014;91(2):133–41. 10.1097/OPX.0000000000000138 24317134

[15] Craig JP, Willcox MDP, Argüeso P, Maissa C, Stahl U, Tomlinson A, et al The TFOS International Workshop on Contact Lens Discomfort: Report of the contact lens interactions with the tear film subcommittee. Investig Ophthalmol Vis Sci. 2013;54(11):TFOS123–56. 24058139 10.1167/iovs.13-13235

[16] Jones L, Brennan N a., González-Méijome J, Lally J, Maldonado-Codina C, Schmidt T a., et al The TFOS International Workshop on Contact Lens Discomfort: Report of the contact lens materials, design, and care subcommittee. Investig Ophthalmol Vis Sci. 2013;54(11):TFOS37–70. 24058138 10.1167/iovs.13-13215

[17] Tran N, Graham AD, Lin MC. Ethnic differences in dry eye symptoms: effects of corneal staining and length of contact lens wear. Cont Lens Anterior Eye. British Contact Lens Association; 2013 12;36(6):281–8. 10.1016/j.clae.2013.06.001 23850062

[18] Niederer RL, McGhee CNJ. Clinical in vivo confocal microscopy of the human cornea in health and disease. Prog Retin Eye Res. Elsevier Ltd; 2010 1;29(1):30–58. 10.1016/j.preteyeres.2009.11.001 19944182

[19] Wise RJ, Sobel RK, Allen RC. Meibography: A review of techniques and technologies. Saudi J Ophthalmol Off J Saudi Ophthalmol Soc. King Saud University; 2012 10;26(4):349–56. 10.1016/j.sjopt.2012.08.007 PMC3729652 23961019

[20] Belmonte C, Garcia-hirschfeld J, Gallar J, Neurociencias I De, Fisiologia D De, Alicante U De. Neurobiology of Ocular Pain. Prog Retin Eye Res. 1996;9462(96):118–49.

[21] Tracey I, Mantyh PW. The cerebral signature for pain perception and its modulation. Neuron. 2007 8 2;55(3):377–91. 17678852 10.1016/j.neuron.2007.07.012

[22] Flor H. Cortical reorganisation and chronic pain: implications for rehabilitation. J Rehabil Med. 2003 10 1;35(4):66–72. 10.1080/16501960310010179 12817660

[23] Villemure C, Bushnell MC. Cognitive modulation of pain: how do attention and emotion influence pain processing? Pain. 2002 2;95(3):195–9. 11839418 10.1016/S0304-3959(02)00007-6

[24] Bushnell MC, Ceko M, Low L a. Cognitive and emotional control of pain and its disruption in chronic pain. Nat Rev Neurosci. 2013 7;14(7):502–11. 10.1038/nrn3516 23719569 PMC4465351

[25] Linton SJ, Shaw WS. Impact of psychological factors in the experience of pain. Phys Ther. 2011 5;91(5):700–11. 10.2522/ptj.20100330 21451097

[26] Green CR, Anderson KO, Baker T a, Campbell LC, Decker S, Fillingim RB, et al The unequal burden of pain: confronting racial and ethnic disparities in pain. Pain Med. 2003 9;4(3):277–94. 12974827 10.1046/j.1526-4637.2003.03034.x

[27] Kil HK, Kim WO, Chung WY, Kim GH, Seo H, Hong J-Y. Preoperative anxiety and pain sensitivity are independent predictors of propofol and sevoflurane requirements in general anaesthesia. Br J Anaesth. 2012 1;108(1):119–25. 10.1093/bja/aer305 22084330

[28] Greenwald HP. Interethnic differences in pain perception. Pain. 1991 2;44(2):157–63. 2052381 10.1016/0304-3959(91)90130-P

[29] Chan MYP, Hamamura T, Janschewitz K. Ethnic differences in physical pain sensitivity: Role of acculturation. Pain. International Association for the Study of Pain; 2013;154(1):119–23. 10.1016/j.pain.2012.09.015 23149393

[30] Ruscheweyh R, Marziniak M, Stumpenhorst F, Reinholz J, Knecht S. Pain sensitivity can be assessed by self-rating: Development and validation of the Pain Sensitivity Questionnaire. Pain. International Association for the Study of Pain; 2009 11;146(1–2):65–74. 10.1016/j.pain.2009.06.020 19665301

[31] Tan E-C, Lim Y, Teo Y-Y, Goh R, Law H-Y, Sia AT. Ethnic differences in pain perception and patient-controlled analgesia usage for postoperative pain. J Pain. 2008 9;9(9):849–55. 10.1016/j.jpain.2008.04.004 18550441

[32] Rahim-williams B, Fillingim RB. A Quantitative Review of Ethnic Group Differences in Experimental Pain Response: Do Biology, Psychology, and Culture Matter? Pain Med. 2012;13(4):522–40. 10.1111/j.1526-4637.2012.01336.x 22390201 PMC3349436

[33] Ruscheweyh R, Verneuer B, Dany K, Marziniak M, Wolowski A, Colak-Ekici R, et al Validation of the Pain Sensitivity Questionnaire in chronic pain patients. Pain. International Association for the Study of Pain; 2012 6;153(6):1210–8. 10.1016/j.pain.2012.02.025 22541722

[34] Granot M. Can we predict persistent postoperative pain by testing preoperative experimental pain? Curr Opin Anaesthesiol. 2009 6;22(3):425–30. 10.1097/ACO.0b013e32832a40e1 19352173

[35] Chapman CR, Donaldson GW, Davis JJ, Bradshaw DH. Improving individual measurement of postoperative pain: the pain trajectory. J Pain. 2011 2;12(2):257–62. 10.1016/j.jpain.2010.08.005 21237721 PMC3052945

[36] Edwards RR. Individual differences in endogenous pain modulation as a risk factor for chronic pain. Neurology. 2005 8 9;65(3):437–43. 16087910 10.1212/01.wnl.0000171862.17301.84

[37] Mobilio N, Gremigni P, Pramstraller M, Vecchiatini R, Calura G, Catapano S. Explaining pain after lower third molar extraction by preoperative pain assessment. J Oral Maxillofac Surg. 2011 11;69(11):2731–8. 10.1016/j.joms.2011.05.023 21835529

[38] Vehof J, Kozareva D, Hysi PG, Harris J, Nessa A, Williams FK, et al Relationship Between Dry Eye Symptoms and Pain Sensitivity. JAMA Ophthalmol. 2013 8 1;1–5. 10.1001/jamaophthalmol.2013.4399 23907167

[39] Kim H-J, Yeom JS, Lee JW, Chang B-S, Lee C-K, Lee G-W, et al The Influence of Pain Sensitivity on the Treatment Outcome of Transforaminal Epidural Steroid Injection in Patients with Lumbar Spinal Stenosis. Pain Pract. 2013 6 5;14(5):405–12. 10.1111/papr.12084 23734752

[40] Kim H-J, Ruscheweyh R, Yeo J-H, Cho H-G, Yi J-M, Chang B-S, et al Translation, Cross-Cultural Adaptation, and Validity of the Korean Version of the Pain Sensitivity Questionnaire in Chronic Pain Patients. Pain Pract. 2013 10 17;1–7. 10.1111/papr.12123 24131768

[41] Kim H-J, Suh B-G, Lee D-B, Park J-Y, Kang K-T, Chang B-S, et al Gender difference of symptom severity in lumbar spinal stenosis: role of pain sensitivity. Pain Physician. 2013;16(6):E715–23. 24284852

[42] Kim H-J, Suh B-G, Lee D-B, Lee G-W, Kim D-W, Kang K-T, et al The influence of pain sensitivity on the symptom severity in patients with lumbar spinal stenosis. Pain Physician. 2013 3;16(2):135–44. 23511680

[43] Hsieh AY, Tripp D a, Ji L-J, Sullivan MJL. Comparisons of catastrophizing, pain attitudes, and cold-pressor pain experience between Chinese and European Canadian young adults. J Pain. Elsevier Inc; 2010 11;11(11):1187–94. 10.1016/j.jpain.2010.02.015 20452836

[44] Svitova TF, Lin MC. Racial variations in interfacial behavior of lipids extracted from worn soft contact lenses. Optom Vis Sci. 2013;90(12):1361–9. 10.1097/OPX.0000000000000098 24270592 PMC3903316

[45] Lin MC, French HM, Graham AD, Sanders TL. Effects of daily irrigation on corneal epithelial permeability and adverse events with silicone hydrogel contact lens continuous wear. Invest Ophthalmol Vis Sci. 2014 2;55(2):776–83. 10.1167/iovs.13-13508 24425854

[46] Lin MC, Yuen J, Graham AD. Contact Lens Care Solutions. Eye Contact Lens Sci Clin Pract. 2014;40(4):191–9. 10.1097/ICL.0000000000000034 PMC4232463 24887209

[47] Lin MC, Yeh TN, Graham AD, Truong T, Hsiao C, Wei G, et al Ocular surface health during 30-day continuous wear: rigid gas-permeable versus silicone hydrogel hyper-O2 transmitted contact lenses. Invest Ophthalmol Vis Sci. 2011 5;52(6):3530–8. 10.1167/iovs.10-6025 21310906

[48] Lin MC, Chen YQ, Polse K a. The effects of ocular and lens parameters on the postlens tear thickness. Eye Contact Lens. 2003;29(1 Suppl):S33–6 –discussion S57–9 –S192–4. 12772727 10.1097/00140068-200301001-00010

[49] Schulz KF, Altman DG, Moher D, Group C. CONSORT 2010 Statement: updated guidelines for reporting parallel group randomised trials. J Clin Epidemiol. Schulz et al; 2010;63(8):834–40. 10.1016/j.jclinepi.2010.02.005 20346629

[50] Peters ML. Emotional and Cognitive influences on pain experience. Mod Trends Pharmacopsychiatry. 2015;30(1):138–52. 26436897 10.1159/000435938

[51] Hart RP, Wade JB, Martelli MF. Cognitive impairment in patients with chronic pain: the significance of stress. Curr Pain Headache Rep. 2003;7(2):116–26. 12628053 10.1007/s11916-003-0021-5

[52] Moriarty O, McGuire BE, Finn DP. The effect of pain on cognitive function: A review of clinical and preclinical research. Prog Neurobiol. Elsevier Ltd; 2011;93(3):385–404. 10.1016/j.pneurobio.2011.01.002 21216272

[53] Karpecki PM. Why Dry Eye Trials Often Fail. Review of Optometry. 2013 1;50–6.

[54] Schaumberg D a, Nichols JJ, Papas EB, Tong L, Uchino M, Nichols KK. The international workshop on meibomian gland dysfunction: report of the subcommittee on the epidemiology of, and associated risk factors for, MGD. Invest Ophthalmol Vis Sci. 2011 3;52(4):1994–2005. 10.1167/iovs.10-6997e 21450917 PMC3072161

[55] Tomlinson A, Bron AJ, Korb DR, Amano S, Paugh JR, Pearce EI, et al The international workshop on meibomian gland dysfunction: report of the diagnosis subcommittee. Invest Ophthalmol Vis Sci. 2011 3;52(4):2006–49. 10.1167/iovs.10-6997f 21450918 PMC3072162

[56] Cuevas M, González-García MJ, Castellanos E, Quispaya R, Parra PD La, Fernández I, et al Correlations among symptoms, signs, and clinical tests in evaporative-type dry eye disease caused by Meibomian gland dysfunction (MGD). Curr Eye Res. 2012 10;37(10):855–63. 10.3109/02713683.2012.683508 22632103

[57] Miller KL, Walt JG, Mink DR, Satram-hoang S, Wilson SE, Perry HD, et al Minimal Clinically Important Difference for the Ocular Surface Disease Index. 2014;128(1):94–101. 10.1001/archophthalmol.2009.356 20065224

[58] Rowell LN, Mechlin B, Ji E, Addamo M, Girdler SS. Asians differ from non-Hispanic Whites in experimental pain sensitivity. Eur J Pain. European Federation of International Association for the Study of Pain Chapters; 2011 8;15(7):764–71. 10.1016/j.ejpain.2010.11.016 21561793 PMC3165029

[59] Lu Q, Tsao J. Multi-Ethnic Differences in Responses to Laboratory Pain Stimuli among Children. Heal Pschology. 2013;32(8):905–14. 10.1037/a0032428 PMC3742024 23668844

[60] Jie Y, Xu L, Wu YY, Jonas JB. Prevalence of dry eye among adult Chinese in the Beijing Eye Study. Eye (Lond). 2009 3;23(3):688–93. 18309341 10.1038/sj.eye.6703101

[61] Galor A, Feuer W, Lee DJ, Florez H, Carter D, Pouyeh B, et al Prevalence and risk factors of dry eye syndrome in a United States veterans affairs population. Am J Ophthalmol. Elsevier Inc.; 2011 9;152(3):377–84.e2. 10.1016/j.ajo.2011.02.026 21684522 PMC4113967

[62] Maïssa C, Guillon M. Tear film dynamics and lipid layer characteristics—effect of age and gender. Cont Lens Anterior Eye. 2010 8;33(4):176–82. 10.1016/j.clae.2010.02.003 20202891

[63] Nien CJ, Massei S, Lin G, Nabavi C, Tao J, Brown DJ, et al Effects of age and dysfunction on human meibomian glands. Arch Ophthalmol. 2011 4;129(4):462–9. 10.1001/archophthalmol.2011.69 21482872 PMC4291168

[64] Pult H, Purslow C, Berry M, Murphy PJ. Clinical tests for successful contact lens wear: relationship and predictive potential. Optom Vis Sci. 2008 Oct;85(10):E924–9. 10.1097/OPX.0b013e3181888909 18832967

[65] Shiraishi A, Yamaguchi M, Ohashi Y. Prevalence of Upper- and Lower-Lid-Wiper Epitheliopathy in Contact Lens Wearers and Non-wearers. Eye Contact Lens Sci Clin Pract. 2014;40(4):220–4. 10.1097/ICL.0000000000000040 24901973

